# Vegetation Types Alter Soil Respiration and Its Temperature Sensitivity at the Field Scale in an Estuary Wetland

**DOI:** 10.1371/journal.pone.0091182

**Published:** 2014-03-07

**Authors:** Guangxuan Han, Qinghui Xing, Yiqi Luo, Rashad Rafique, Junbao Yu, Nate Mikle

**Affiliations:** 1 Key Laboratory of Coastal Environmental Processes and Ecological Remediation, Yantai Institute of Coastal Zone Research, Chinese Academy of Sciences, Yantai, China; 2 Department of Microbiology and Plant Biology, University of Oklahoma, Norman, Oklahoma, United States of America; Beijing Forestry University, China

## Abstract

Vegetation type plays an important role in regulating the temporal and spatial variation of soil respiration. Therefore, vegetation patchiness may cause high uncertainties in the estimates of soil respiration for scaling field measurements to ecosystem level. Few studies provide insights regarding the influence of vegetation types on soil respiration and its temperature sensitivity in an estuary wetland. In order to enhance the understanding of this issue, we focused on the growing season and investigated how the soil respiration and its temperature sensitivity are affected by the different vegetation (*Phragmites australis*, *Suaeda salsa* and bare soil) in the Yellow River Estuary. During the growing season, there were significant linear relationships between soil respiration rates and shoot and root biomass, respectively. On the diurnal timescale, daytime soil respiration was more dependent on net photosynthesis. A positive correlation between soil respiration and net photosynthesis at the *Phragmites australis* site was found. There were exponential correlations between soil respiration and soil temperature, and the fitted *Q*_10_ values varied among different vegetation types (1.81, 2.15 and 3.43 for *Phragmites australis*, *Suaeda salsa* and bare soil sites, respectively). During the growing season, the mean soil respiration was consistently higher at the *Phragmites australis* site (1.11 µmol CO_2_ m^−2^ s^−1^), followed by the *Suaeda salsa* site (0.77 µmol CO_2_ m^−2^ s^−1^) and the bare soil site (0.41 µmol CO_2_ m^−2^ s^−1^). The mean monthly soil respiration was positively correlated with shoot and root biomass, total C, and total N among the three vegetation patches. Our results suggest that vegetation patchiness at a field scale might have a large impact on ecosystem-scale soil respiration. Therefore, it is necessary to consider the differences in vegetation types when using models to evaluate soil respiration in an estuary wetland.

## Introduction

Vegetation type plays an important role in regulating the temporal and spatial variations of soil respiration through controlling a variety of environmental variables [1]–[4]. These variables can be grouped into (1) the amount and chemical composition of organic matter deposited onto the soil surface [3], [5], [6], (2) the amount of plant C allocate to belowground [7]–[9], (3) the amount of photoassimilates that fuel the metabolic processes of mycorrhizae, endophytes and microbial populations in the rhizosphere [10], [11], (4) soil microbial communities [12]–[14], and (5) soil properties and near-surface atmosphere [4], [9], [15], [16]. Consequently, it is a key prerequisite to understand the impacts of vegetation on soil respiration for an accurate prediction of the future terrestrial carbon balance in the context of climate change [2], [9].

In ecosystems with steep physical/chemical gradients (e.g., estuarine salinity gradients, water gradients in arid and semi-arid ecosystems), vegetation distribution is markedly patchy and shows mosaic patterns [5], [6], [17], [18]. Vegetation patches may modify microenvironment and resource availability, by altering the dynamics of biomass, organic matter and nutrients [6], [17], [19]–[21], with significant consequences for soil respiration processes at several different scales [2], [8], [20]. For instance, at the plot scale, there is a gradient in root density and root respiration with increasing distance from the trunk [22], [23]. At the field scale, vegetation plays an important role in nutrient cycling [17]. Soil respiration from vegetated patches is larger than from bare soil [6]–[8]. At the ecosystem scale, forest and plant community composition affected soil respiration by controlling substrate quantity and quality, and environmental conditions [1], [15], [21]. In addition, the temperature sensitivity of soil respiration (i.e. *Q*_10_) is also regulated by vegetation properties, including plant photosynthesis and productivity [24], [25], plant phenology [26], [27], quality and quantity of root exudates [28], [29]. Consequently, vegetation patchiness may introduce uncertainties in the estimates of the soil respiration models designed for scaling up field measurements to ecosystem levels [3], [30].

**Figure 1:**
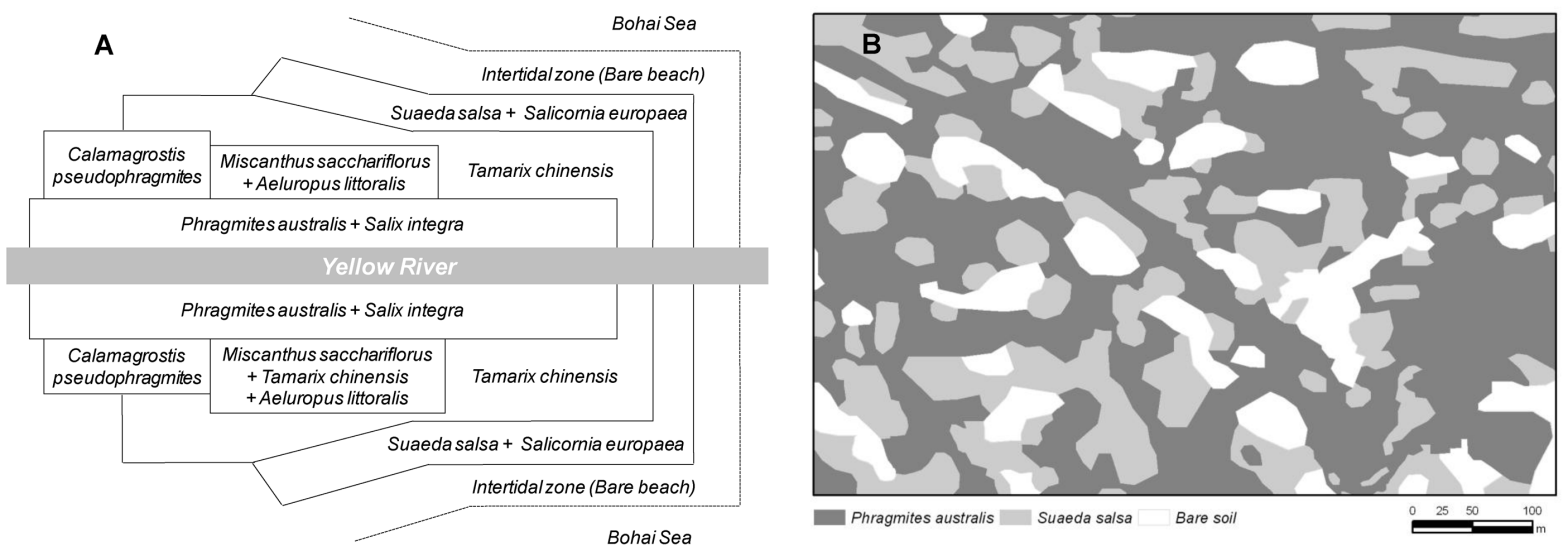
Spatial distribution patterns of wetland vegetation at different spatial scales in the Yellow River Estuary. (A), At the landscape and regional scale (from several kilometers to tens of kilometers), various vegetation types develop and wriggle upwards along with the river pathway from the sea to the land (Modified from Fang [33]); (B), At the field scale (from several meters to tens of meters), the spatial distribution patterns of vegetation are mostly identified as patches of *Phragmites australis*, *Suaeda salsa* or bare soil in many sites.

The Yellow River Estuary is one of the most active regions of land-ocean interaction among the large river deltas in the world. Water and sand sedimentation from the Yellow River forms an important base for the extension and development of the Yellow River Delta [31]. Meanwhile, sea water intrusion continuously erodes the newly formed land [32]. Under the combined action between freshwater and seawater, as well as between groundwater and surface water, the spatial distribution patterns of vegetation are mostly identified as patches or strips of salt-tolerant herbs, grasses, and shrubs along soil moisture-salinity gradients [31]–[34]. At the landscape and regional scale (from several kilometers to tens of kilometers), various vegetation types develop and wriggle upwards along with the river pathway from the sea to the land across horizontal estuarine salinity gradients (Fig. 1A) [31], [33]. In addition, bare patches occur where soil salinity is too high for plants to grow. As a result, at the field scale (from several meters to tens of meters), the spatial distribution patterns of vegetation are sometimes identified as patches of salt tolerant herbs, grasses, shrubs with relatively dense cover separated by more or less bare soil (Fig. 1B). These differences among vegetation types in physiology and phenology may play major roles in determining soil respiration and its temperature sensitivity.

To our knowledge, very few studies have been conducted on the influence of vegetation types on soil respiration and its temperature sensitivity in an estuary wetland. In this study, we sought to examine the relation between vegetation patch types and soil respiration in three adjacent vegetation types, *Phragmites australis*, *Suaeda salsa* and bare soil, in the Yellow River Estuary. Our objectives were to (1) examine seasonal patterns and spatial variation of soil respiration in the three adjacent vegetation types (*Phragmites australis*, *Suaeda salsa* and bare soil); (2) evaluate the impact of vegetation types on the spatial variation of soil respiration at a field scale in the estuary ecosystem, and (3) quantify the effects of vegetation types on the temperature sensitivity of soil respiration during the growing season.

## Materials and Methods

### Ethics Statement

We carried out the study at Yellow River Delta Ecological Research Station of Coastal Wetland, which belongs to the Yantai Institute of Coastal Zone Research, Chinese Academy of Sciences. All necessary permits were obtained for the described field study. We confirm that our study had no harm to the environment and the field studies did not involve endangered or protected species.

### Site Description

The study sites are located at Yellow River Delta Ecological Research Station of Coastal Wetland (37°45′50 ″N, 118°59′24″E), Chinese Academy of Sciences, in Kenli County, Shandong Province, China. The climate is dominated by a warm-temperate and continental monsoon with distinctive seasons and a rainy summer. The annual mean air temperature and annual precipitation are 12.9°C and 560 mm, respectively. More than 50% of the annual precipitation occurs from July to September. The periodic surface ponding is often observed in the estuary wetlands, following heavy rainfall events. The soil type gradually varies from fluvo-aquic to saline soil. The soil texture is mainly identified as sandy clay loam. The most widespread vegetation types in the Yellow River Estuary are *Phragmites australis*, *Suaeda salsa*, *Tamarix chinensis*, and *Imperata cylindrical* var. major. In our research sites, the spatial distribution patterns of vegetation are mostly identified as patches of *Phragmites australis*, *Suaeda salsa* or bare soil on scales of meters to tens of meters because of the obvious gradient change of soil salt content. The main growing season of the estuary wetland ecosystem ranges from late April to late October.

### Soil Respiration and Microclimate Measurements

Three different vegetation sites (*Phragmites australis*, *Suaeda salsa* and bare soil) were established, each one was 15 m×15 m. The three sites were located no more than 30 m apart (Fig. 1B). Three sampling points at each site were selected for soil respiration measurements. Soil collars with a height of 11.4 cm and diameter of 21.3 cm were inserted 7 cm into the soil one week before the first measurement. Living weeds inside the collars were carefully clipped from the soil surface. The soil collars were left in place throughout the entire study period. Soil respiration was measured by a soil CO_2_ efflux measurement system (LI-8100, Licor, Lincoln, NE, USA). The chamber was closed for 120 s and the linear increase of CO_2_ concentration in the chamber was used to estimate soil respiration every 15 days during the growing season. The measurements were taken every 2 h from 7∶00 to 19∶00 (local time) at clear days. For each site, seven measurements were performed each day.

### Photosynthesis and Meteorological Measurements

In order to evaluate the effect of photosynthesis on soil respiration, the daily course of photosynthesis was also measured simultaneously with soil respiration measurements at the *Phragmites australis* site. Using a LI-6400 XT infrared gas analyzer (Licor, Lincoln, NE, USA), the rate of photosynthesis was measured at 2 h intervals from 7∶00 to 19∶00 on May 15, May 27, June 12 and July 9. All measurements were conducted *in situ* on the upper most fully expanded leaves of 5 individuals of *Phragmites australis*. The leaves of *Suaeda salsa* are line-shaped and fleshy, but there is no instrumentation for monitoring photosynthetic rates of fleshy leaves. Thus, the photosynthesis rates of *Suaeda salsa* were not measured in our present study.

During each soil respiration measurement, meteorological parameters were measured simultaneously with an array of sensors, including net radiation (CNR4, Kippand Zonen USA Inc., Bohemia, NY, USA), air temperature (HMP45C, Vaisala, Helsinki, Finland), and precipitation (TE525 tipping bucket gauge, Texas Electronics, Texas, USA). Soil temperature (every 10 cm from 0 to 50 cm depth) was measured with thermistors (109SS, Campbell Scientific Inc., USA). Soil water content was measured by time domain reflectometry probes (EnviroSMART SDI-12, Sentek Pty Ltd., USA) at 10 cm, 20 cm and 40 cm depths below the surface. These data were logged every 30 min by a CR1000 datalogger (Campbell Scientific Inc., USA).

### Soil and Vegetation Sampling and Analysis

On each site of soil respiration measurement, every month we randomly selected six soil samples in each vegetation types to measure soil properties, including soil organic carbon (SOC), total carbon (C), and total nitrogen (N). SOC was determined using wet combustion, total soil C was analyzed using the potassium dichromate oxidation method, and total soil N was measured by the Kjeldahl method. In addition, the shoot and root biomass of *Phragmites australis* and *Suaeda salsa* communities were measured by harvesting the vegetation approximately every 2 weeks. Five 0.5 m×0.5 m squares were randomly chosen in the *Phragmites australis* and *Suaeda salsa* communities, respectively. Live plants were clipped at 1 cm above the ground level, and root biomass was measured by taking five soil blocks (0.5 m wide×0.5 m long×0.3 m deep). Shoot and root biomass were oven dried at 80°C for 48 h and weighed.

### Data Analysis

Linear regression and correlation analysis were used to quantify the effect of shoot and root biomass on seasonal variations in soil respiration rate at *Phragmites australis* and *Suaeda salsa* sites. In the *Phragmites australis* community, correlation analysis was used to evaluate the effect of net photosynthesis rate on soil respiration during daytime on May 15, May 27, June 12 and July 9, respectively.

Exponential regression analysis between soil respiration and temperature used the data of soil respiration and soil temperature at 10 cm depth in the 3 different vegetation patches, respectively:
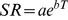
(1)where *SR* is soil respiration, *T* is soil temperature, coefficient *a* is the intercept of soil respiration when temperature is zero, and coefficient *b* represents the temperature sensitivity of soil respiration.

*Q*_10_ can be estimated as
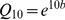
(2)


Data were first tested for normality and homogeneity of variance, then one-way analysis of variance (ANOVA) was used to test for significant differences of soil respiration, shoot biomass, root biomass, litter biomass, total C, total N and SOC at 0–20 cm depth among three vegetation patches (*Phragmites australis*, *Suaeda salsa* and bare soil) with Tukey’s studentized range test using α = 0.05 for significance. In order to evaluate the impact of vegetation types on the spatial variation of soil respiration, the relationship between soil respiration and biotic factors (shoot, root and litter biomass) and soil chemical properties (SOC, total C and total N) were examined using correlations matrixes, and the Pearson’s coefficient was used to establish correlations between each pair of variables. All statistical analyses were performed using SPSS 11.5 (SPSS for Windows, Version 11.5, Chicago, IL, USA).

## Results

### Meteorological Conditions and Plant Biomass

**Figure 2:**
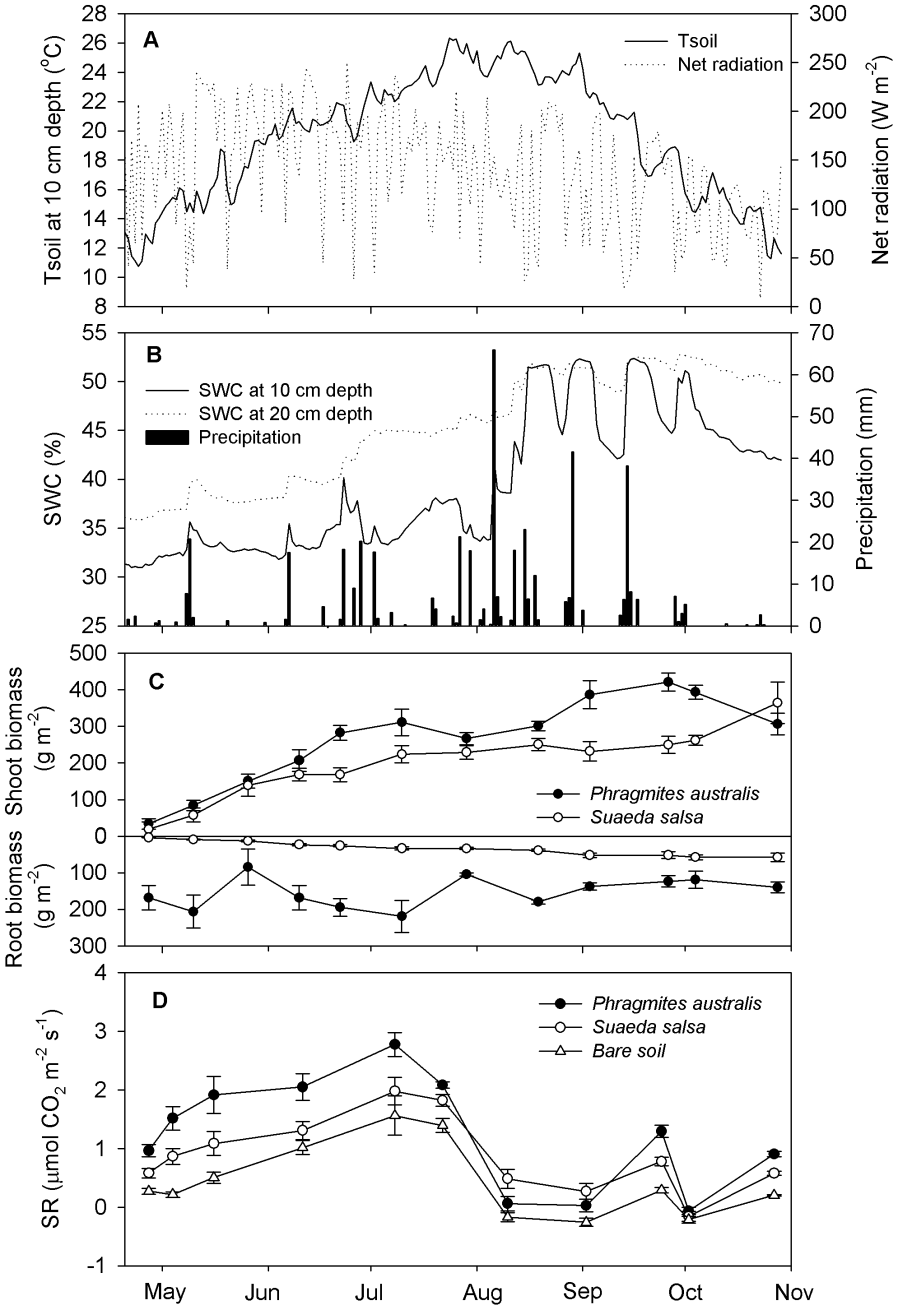
Temporal patterns of soil respiration and environmental factors among sites. (A), daily means of net radiation and soil temperature (Tsoil) at 10 cm depth; (B), daily means of soil water content (SWC) at 10 cm and 20 cm depth and precipitation; (C), averaged shoot and root biomass of *Phragmites australis* and *Suaeda salsa*; (D), daily means of soil respiration (SR). Error bars represent ± SE.

The seasonal variations of net radiation (Rn), soil temperature, soil water content (SWC), precipitation, and plant biomass are shown in Fig. 2. The distribution of Rn showed a large variation with a daily maximum (250.5 W m^−2^) in late June and a minimum (8.1 W m^−2^) in late October (Fig. 2A). During the growing season, the daily average soil temperature ranged from 10.7 to 26.3°C. The maximum soil temperature was recorded in late July to early August (Fig. 2A). Precipitation concentrated in the period of August to September, with the largest daily rainfall of 65.9 mm occurred on 6 August (Fig. 2B). Seasonal fluctuations in precipitation affected SWC at the 10-cm and 20-cm depths (Fig. 2B).

At *Phragmites australis* and *Suaeda salsa* sites, the shoot biomass increased during the early growing season, but their seasonal variation patterns differed markedly between the two sites (Fig. 2C). The shoot biomass of *Phragmites australis* and *Suaeda salsa* reached their maximum in late September (420.5 g m^−2^) and late October (363.8 g m^−2^), respectively (Fig. 2C). During the growing season, the root biomass of the *Phragmites australis* community (153.9 g m^−2^) was significantly higher than that of the *Suaeda salsa* community (33.4 g m^−2^) (*P*<0.001, Fig. 2C).

### Seasonal Variation of Soil Respiration

The seasonal variations of soil respiration at *Phragmites australis*, *Suaeda salsa* and bare soil sites showed similar patterns with higher rates in July when soil temperature or plant biomass were higher, and with lower values during the surface ponding period (Fig. 2). From late April, soil respiration increased gradually as the soil temperature increased and the plant grew, and then increased to a peak in mid-July. Subsequently, soil respiration rates followed a steady decrease towards October. The negative values of soil respiration occurred during August and September when surface ponding occurred. Throughout the measured period, soil respiration at the *Phragmites australis*, *Suaeda salsa* and bare soil sites ranged from -0.07 to 2.78 µmol CO_2_ m^−2 ^s^−1^, -0.16 to 1.98 µmol CO_2_ m^−2 ^s^−1^, and -0.26 to 1.56 µmol CO_2_ m^−2 ^s^−1^, respectively. Additionally, the coefficients of variation (CVs) for soil respiration at the *Phragmites australis*, *Suaeda salsa* and bare soil sites were 77.2%, 73.5%, and 143.5%, respectively, over the whole measurement period.

### Effect of Shoot and Root Biomass on Seasonal Variation of Soil Respiration

**Figure 3:**
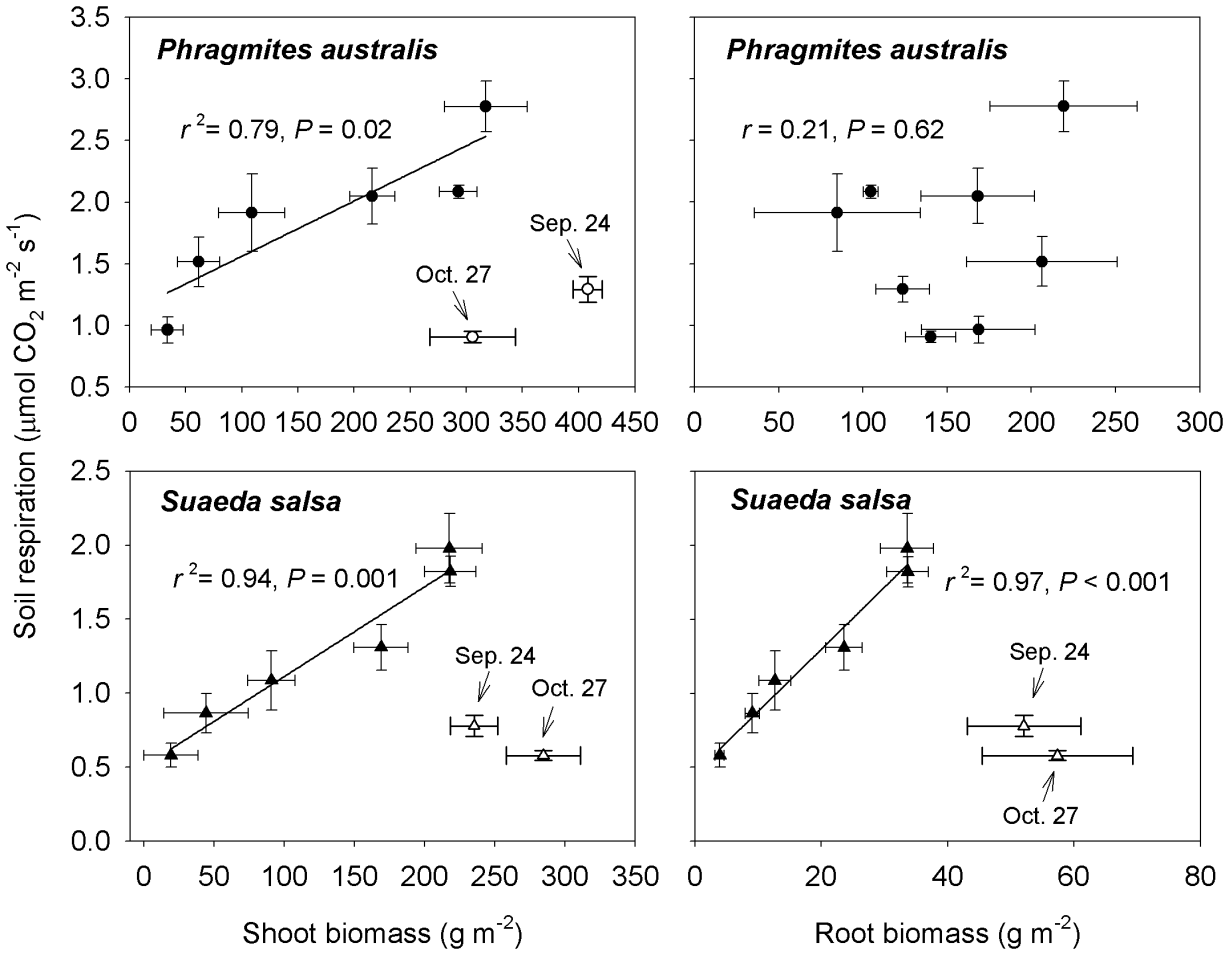
Relationships between mean soil respiration and plant biomass at *Phragmites australis* and *Suaeda salsa* sites. Bars represent standard errors of the means.

At the *Phragmites australis* site, soil respiration rates showed no significant correlations with shoot and root biomass (*r* = 0.22 and *P* = 0.61, and *r* = 0.21 and *P* = 0.48, respectively). However, during the early and peak growing season (from late April to late July), there was a good linear relationship between soil respiration and shoot biomass (Fig. 3A, *r* ^2^ = 0.79, *P = *0.02). Similarly, at the *Suaeda salsa* site, there was no significant relationship between soil respiration and shoot and root biomass during the entire growing season. However, during the initial growing season, there were significant linear relationships between soil respiration rates and shoot biomass (Fig. 3C, *r* ^2^ = 0.94, *P = *0.001) and root biomass (Fig. 3D, *r* ^2^ = 0.97, *P*<0.001), respectively.

### Effect of Net Photosynthesis on Diurnal Variation of Soil Respiration

**Figure 4:**
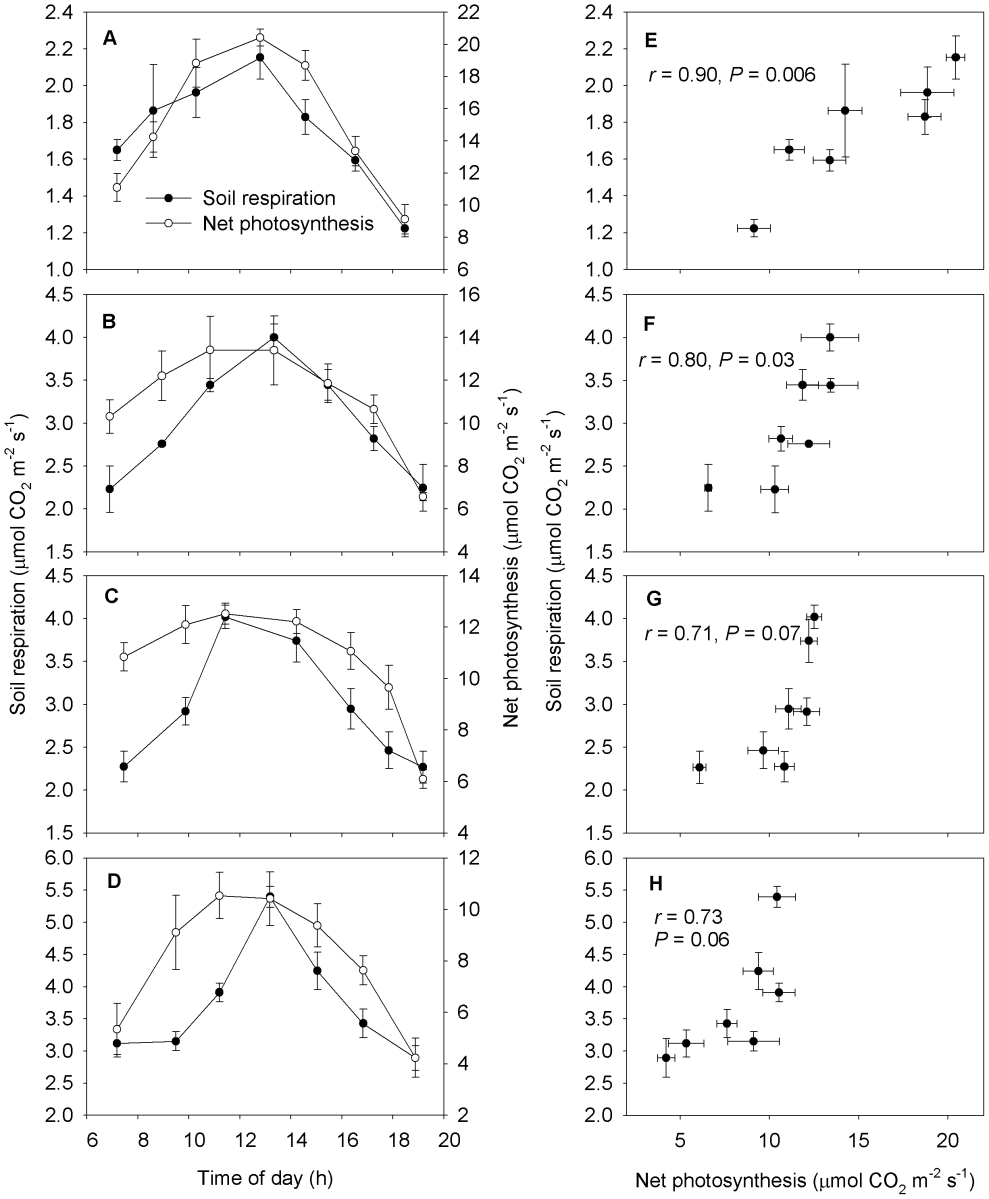
Diurnal variations in soil respiration and net photosynthesis and their relationships at *Phragmites australis* community. May 15 (A, E), May 27 (B, F), June 12 (C, G) and July 9 (D, H). Bars represent standard errors of the means.

In the *Phragmites australis* community, the diurnal variation of net photosynthesis showed a bell shape with the peak almost exactly at noon on May 15, May 27, June 12 and July 9, respectively (Fig. 4A, B, C, and D). The diurnal fluctuations of soil respiration varied correspondingly with net photosynthesis and showed asymmetric patterns with the maximum between 12∶00–14∶00 h. In the morning hours, soil respiration increases gradually coinciding with increasing net photosynthesis levels. In the afternoon hours, soil respiration decreased slightly following the decreasing trend of net photosynthesis. There was a significant positive correlation between soil respiration and net photosynthesis on May 15 (Fig. 4E, *r* = 0.90, *P = *0.006) and May 27 (Fig. 4F, *r* = 0.80, *P = *0.03), respectively. In addition, the soil respiration rates showed no significant correlations with net photosynthesis rates on June 12 (Fig. 4G, *r* = 0.71, *P = *0.07) and July 9 (Fig. 4H, *r* = 0.73, *P = *0.06).

### Temperature Sensitivity of Soil Respiration among Different Vegetation Types

**Figure 5:**
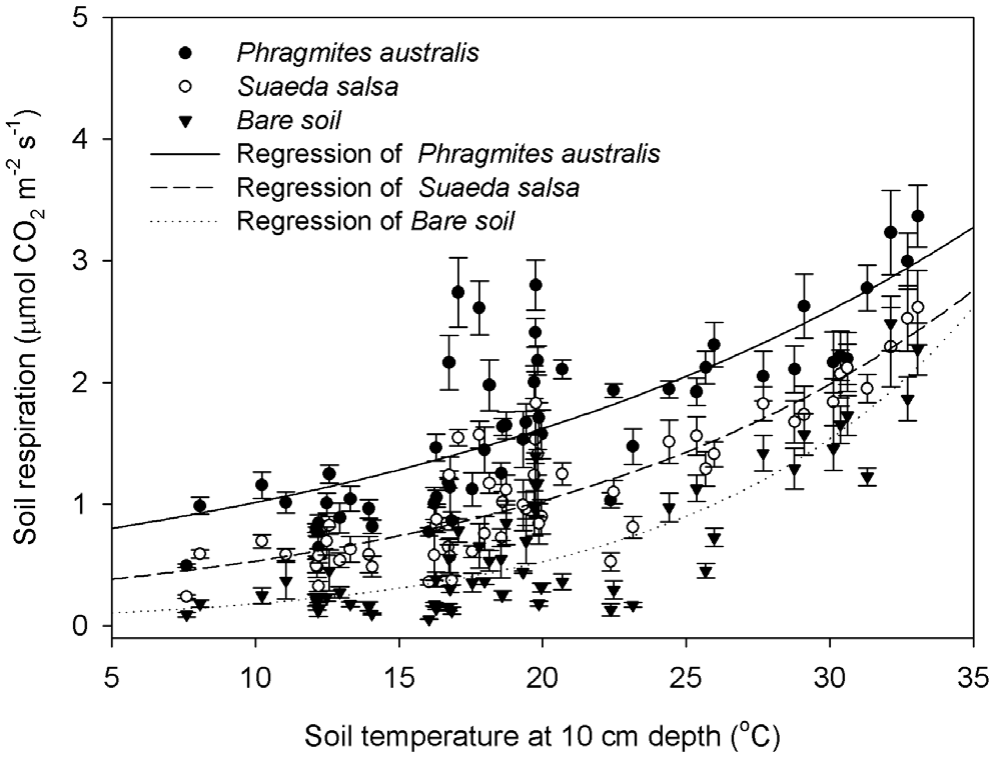
Responses of soil respiration to soil temperature during the growing season among sites. Black lines are the best fits. Bars represent standard errors of the means.

Many studies usually analyzed the temperature sensitivity of soil respiration using soil temperature at a shallow depth [24], [29], [35]. Thus, in this study we selected soil temperature at 10 cm to investigate the influence of temperature on soil respiration. There were significant exponential relationships between soil respiration and soil temperature during the growing season for the *Phragmites australis* (*r* ^2^ = 0.62, *P*<0.001), *Suaeda salsa* (*r* ^2^ = 0.77, *P*<0.001) and bare soil site (*r* ^2^ = 0.75, *P*<0.001), respectively (Fig. 5 and Table 1). The temperature sensitivity coefficient *a* (basal respiration) of Eq. (1) for the *Phragmites australis* (0.47±0.15) was significantly greater (*P*<0.05) than those for the *Suaeda salsa* (0.22±0.01) and the bare soil (0.04±0.01). No significant difference in basal respiration between the *Phragmites australis* and bare soil sites was detected (*P*>0.05; Table 1). The temperature sensitivity coefficient *b* of Eq. (1) at the bare site (0.12±0.01) was higher (*P*<0.05) than that at the *Phragmites australis* (0.06±0.01) and *Suaeda salsa* (0.08±0.01) sites (Table 1). Consequently, *Q*_10_ (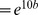) at bare soil site (3.43±0.17) was significantly higher (*P*<0.05) than vegetated sites (1.81±0.11 for the *Phragmites australis* and 2.15±0.10 for the *Suaeda salsa*). However, there was no significant difference in the *Q*_10_ values between the *Phragmites australis* and *Suaeda salsa* sites (*P*>0.05; Table 1).

**Table 1:** Values of coefficients *a* and *b* of the Eq. (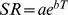), the temperature sensitivity of soil respiration (*Q*_10_) and their one-way ANOVA test among different vegetation patches during the growing season in a estuary wetland.

Vegetation patches	*a*	*b*	*Q*_10_	*r* ^2^	*n*	*P*
*Phragmites australis*	0.47±0.15 a	0.06±0.01 a	1.81±0.11 a	0.62	56	<0.001
*Suaeda salsa*	0.22±0.01 b	0.08±0.01 a	2.15±0.10 a	0.77	56	<0.001
Bare soil	0.04±0.01 b	0.12±0.01 b	3.43±0.17 b	0.75	56	<0.001

*a*, *b* are coefficients of the Eq. (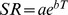), *Q*_10_ is the temperature sensitivity of soil respiration (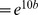), *r* ^2^ is the determinant coefficient. n is the number of samples data. Numbers in brackets represent the standard error of the mean. A one-way ANOVA was used to compare *a*, *b*, and *Q*_10_ values among different vegetation patches (n = 3). Different letters indicate significant difference (*P*<0.05) among different vegetation patches.

### Spatial Variations of Soil Respiration and Its Environmental Factors

**Figure 6:**
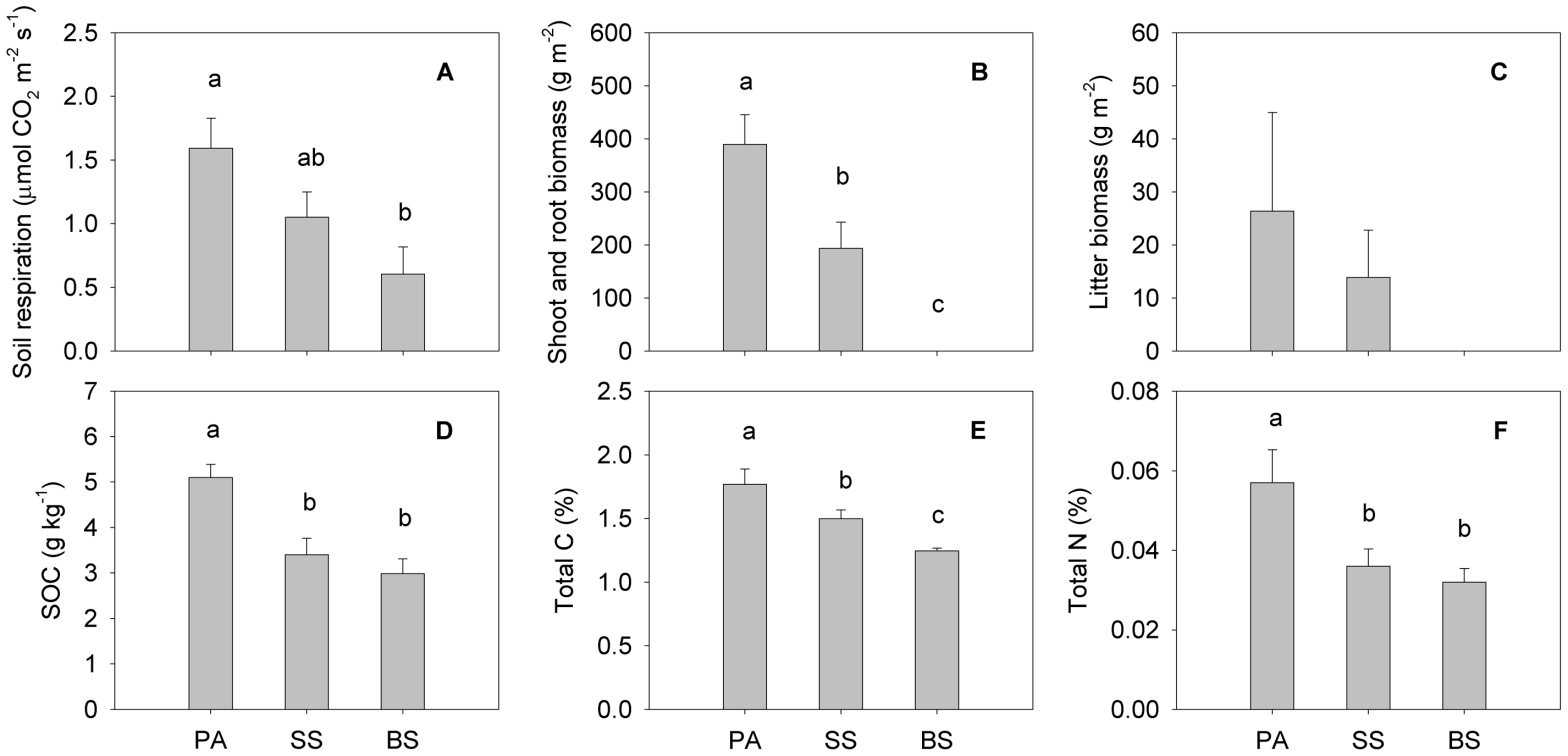
Spatial variations of soil respiration and environmental factors among sites. Average soil respiration rates (A), shoot and root biomass (B), litter biomass (C), SOC (D), total C (E), and total N (F) of *Phragmites australis* (PA), *Suaeda salsa* (SS) and bare soil (BS) patches during the growing season in the Yellow River Estuary, China. Standard error of each mean is represented over each bar. Different letters denote significant (*P*<0.05) differences among the three sites (Tukey test after one way-ANOVA).

During the growing season, the average soil respiration at the *Phragmites australis*, *Suaeda salsa* and bare soil sites was 1.59, 1.05 and 0.60 µmol CO_2_ m^−2^ s^−1^, respectively. The mean soil respiration of the *Phragmites australis* community was significantly higher than that at the bare soil site (*P*<0.05), whereas no significant differences in the soil respiration could be found neither between the *Suaeda salsa* community and the *Phragmites australis* community or between the *Suaeda salsa* community and the bare soil site (*P*>0.05, Fig. 6A). Soil respiration at the bare soil site was 37.7% and 66.0% of the *Phragmites australis* and *Suaeda salsa* communities, respectively. The biomass of *Phragmites australis* was significantly higher compared with that of *Suaeda salsa* (*P*<0.05, Fig. 6B). However, no significant difference in litter biomass was observed (*P*>0.05) among the three vegetation patches (Fig. 6C). Furthermore, the *Phragmites australis* community had significantly higher SOC, total C, and total N than that of the *Suaeda salsa* community and the bare soil site (Fig. 6D, E, and F). Consistently for all sites, the best factors explaining the site-to-site variation of soil respiration may be the plant biomass and soil properties.

### Effect of Vegetation Types on Spatial Variation of Soil Respiration

**Figure 7:**
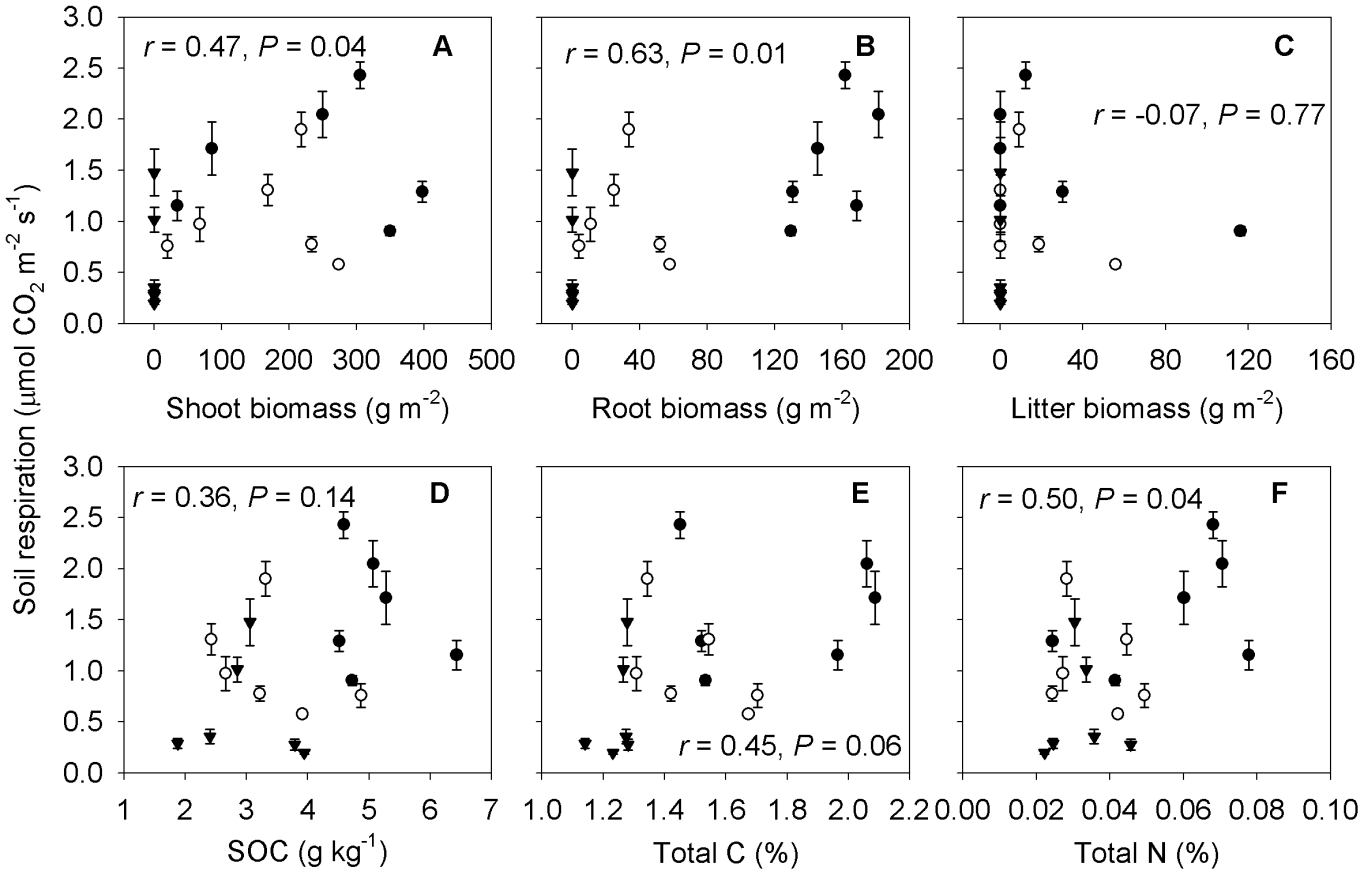
Relationships between average monthly soil respiration and environmental factors among sites. Shoot biomass (A), root biomass (B), litter biomass (C), SOC (D), total C (E), and total N (F) of three adjacent vegetation types (*Phragmites australis*, *Suaeda salsa* and bare soil sites). Bars represent standard errors of the means. One point represents the average soil respiration and average environmental factors of each patch during one month of measurement. Closed circles (•) represent *Phragmites australis* community, open circles (○) represent *Suaeda salsa* community, and closed triangles (▴) represent bare soil site.

The mean monthly soil respiration correlated positively with shoot biomass (*r = *0.47, *P* = 0.04; Fig. 7A) and root biomass (*r = *0.63, *P* = 0.01; Fig. 7B), among the *Phragmites australis*, *Suaeda salsa* and bare soil sites respectively. No significant correlation could be found between the mean monthly soil respiration and litter biomass among the three vegetation patches (*r* = -0.07, *P* = 0.77; Fig. 7C). Similarly, there was no significant correlation between the mean soil respiration and SOC at 0–20 cm depth (*r = *0.36, *P* = 0.14; Fig. 7D). However, the mean soil respiration increased with an increase in total C at 0–20 cm depth (*r = *0.45, *P* = 0.06; Fig. 7E) and total N at 0–20 cm depth (*r = *0.50, *P* = 0.04; Fig. 7F) among the three vegetation patches.

## Discussion

### Biotic Factors Drive the Temporal Variation of Soil Respiration

On the diurnal timescale, daytime soil respiration varied correspondingly with net photosynthesis. A positive correlation between soil respiration and net photosynthesis at the *Phragmites australis* site was observed (Fig. 4). These results are consistent with previous findings showing that diurnal changes in the supply of newly produced photosynthates regulate soil respiration. For instance, the diurnal pattern of root-derived CO_2_ efflux and induced rhizosphere priming effects are coupled with the plant photosynthetic cycle [36]. Daytime soil respiration showed significant responses to plant photosynthetic activity with a time lag of about 0–3 h in the steppe ecosystem [37]. Generally, photosynthesis may modulate respiration in multiple ways [13], [38]. Firstly, the root and rhizosphere respiration is tightly coupled with aboveground photosynthesis and is largely controlled by allocation of recent photosynthates to belowground [15], [36], [39]. Secondly, the flux of recent photosynthate supports substantial microbial activity in the rhizosphere, which can in turn influence the relative fraction of heterotrophic respiration [12], [13]. Thirdly, the respiration of mycelium has also been found to be dependent on newly produced photosynthates [40]. Thus, soil respiration is directly driven by recent photosynthesis [41]–[43]. Consequently, photosynthesis rates might be different among different vegetation types, which may lead to variation in soil respiration rates even under the same soil temperature and moisture.

During the early and peak growing season of *Phragmites australis* and *Suaeda salsa* communities, there were significant linear relationships between soil respiration rates and shoot biomass and root biomass, respectively (Fig. 3). The results are in line with previous studies suggesting that soil respiration is directly related to plant biomass [26], [44], [45]. A number of studies have demonstrated that besides soil temperature and soil moisture, changes in plant biomass may also contribute to temporal variations in soil respiration in several ways. On the one hand, the increase in aboveground production implied an increase in total root carbon allocation, thus enhanced root respiration [26], [46]. On the other hand, aboveground biomass could supply the inputs to the soil of aboveground litter and belowground organic detritus, and consequently dictated heterotrophic soil respiration [47]. In addition, root biomass below the measurement chambers probably directly influenced soil respiration rates [44] since root respiration comprise 40–60% of total soil respiration [48], although these values also strongly depended on the vegetation growth stage. Therefore, increased plant biomass growth presumably enhances root and microbial respiration, by affecting carbon allocation and substrate supply for plant roots and soil microbes in rhizosphere [23], [26], [49], thus contributing to soil respiration. As such, any activity that modifies vegetation productivity and biomass could have quantitative effects on soil respiration. However, over the whole growing season, the soil respiration rates showed no correlations at both vegetated sites with shoot and root biomass (*P*>0.05). Plant growth itself is also under the influence of temporal changes in soil temperature and moisture. Consequently, temporal changes in soil temperature and moisture are likely to mask the effects of plant biomass and photosynthesis on soil respiration [16].

### Vegetation Types Alter the Temperature Sensitivity of Soil Respiration

The relationships between soil respiration and soil temperature were best described by exponential equations at each site (Fig. 5 and Table 1), which have been widely demonstrated in other ecosystems [7], [16], [44]. In addition, a negative soil respiration occurred during surface ponding (Fig. 1), which is worth of attention and further research to explain the phenomenon. Excessive soil moisture can lead to decreases in soil respiration by reducing the oxygen supply for both microbial decomposition and autotrophic activities [50]. Parsons et al. [51] suggested that the negative soil respiration may be caused by the influence of physical factors on inorganic C, including temperature influences on the solubility of CO_2_ and carbonate dissolution chemistry. Ball et al. [52] hypothesized that changes in temperature, soil moisture and other physical factors control the magnitude of negative soil CO_2_ fluxes. Increasing soil moisture allowed more CO_2_ to be dissolved in the soil solution, and when coupled with decreasing temperature, the largest negative CO_2_ flux rates were observed [52].

The *Q*_10_ of soil respiration was found to vary among different vegetation types in this study. The *Q*_10_ value of the bare soil (3.43) was significantly greater than that in two vegetated sites (1.81 for the *Phragmites australis* community and 2.15 for the *Suaeda salsa* community), which means that soil organic matter in bare soil is most sensitive to changes in soil temperature. Increasing evidence suggests that the apparent temperature response of soil respiration is closely related to vegetation type and activity. For example, Zheng et al. [35] found that ecosystems of different vegetation types showed different *Q*_10_ values of soil respiration in 49 ecosystems in China. By analyzing 384 field measurement data, Wang et al. [15] demonstrated that the seasonal amplitude of satellite vegetation greenness index observations (NDVI) was significantly and positively correlated with the apparent *Q*_10_ of soil respiration.

Among the vegetation types observed in this study, the relative proportions of temperature-sensitive and -insensitive respiration fluxes among soil respiration might be different. Some studies have found that the mycorrhizal respiration, root respiration and the plant growth respiration are largely independent of temperature [10], [53], [54], because the growth respiration is mostly associated with the C supply from aboveground [13], [38], [55]. Therefore, microbial respiration is more sensitive than root and rhizosphere respiration to soil temperature [42]. At the bare soil site, the relative contribution from microbial respiration to soil respiration might be larger compared to the two vegetated sites, causing higher value of *Q*_10_ at the bare soil site. Moreover, the temperature sensitivity of the organic matter should increase as the quality of the substrate decreases [56], which has been supported by experimental evidence [28], [29]. Compared with vegetated sites, there was a significant reduction in quality of the photosynthates from the root and rhizosphere at the bare soil site, which would increase *Q*_10_ of soil respiration. Under optimum soil water conditions, *Q*_10_ decreased with increasing gross primary production (GPP), which could also be due to improved quality of substrate arising from increased supply of recent photosynthates [29].

Meanwhile, among the different vegetation types, contribution of soil respiration components (microbial and root respiration) to the overall soil CO_2_ efflux might be different, which may differentially regulates the temperature sensitivity of soil respiration. However, disagreement exists over the relative temperature sensitivities of microbial and root respiration, since the two components are difficult to partition *in situ*. Several contradictory results have suggested that the root respiration should be more sensitive to changes in temperature [24], [25]. There is evidence to suggest that a small deviation of *Q*_10_ may cause a significant bias in the estimate of soil respiration [26], [50]. Therefore it will be necessary to further investigate the effect of vegetation types on the apparent *Q*_10_ of soil respiration at the regional scale studies.

### Vegetation Types Alter Spatial Variation of Soil Respiration

During the growing season, the mean soil respiration was consistently the highest at the *Phragmites australis* site (1.59 µmol CO_2_ m^−2^ s^−1^), followed by the *Suaeda salsa* site (1.05 µmol CO_2_ m^−2^ s^−1^) and the bare soil site (0.60 µmol CO_2_ m^−2^ s^−1^). The differences in vegetation-related controls on soil respiration have been evaluated in different places for different ecosystems [20], [57]. Whereas soil temperature and moisture are the most influential environmental factors controlling the temporal variation of soil respiration, they are inadequate to explain the spatial variations of soil respiration within a site and between sites [23], [50], [58]. In this study, the three sampling sites were contiguous to each other and experienced similar temperature and precipitation patterns, therefore, the differences in vegetation types or soil properties might drive the site difference in soil respiration.

The mean monthly rate of soil respiration was positively correlated with shoot and root biomass (Fig. 7A, B), which is in agreement with several recent studies suggesting that plant biomass is the dominant control on the spatial variation of soil respiration [44], [59], [60]. Among six forest sites with different primary succession stages, the mean soil respiration rate positively correlated with plant biomass [21]. In alpine grasslands along a transect across the Tibetan Plateau, most (80%) of the variation in soil respiration could be attributed to the difference in belowground biomass among 42 sites [45]. In our study, the bare soil site had the lowest soil respiration among the 3 sites, which is largely because there was almost no shoot or root biomass (Fig. 6B, C).

Besides the influence of biotic factors, soil chemical properties in the present study showed large spatial variation among the three vegetation types (Fig. 6D, E and F). The mean soil respiration was positively correlated with total N and total C at 0–20 cm depth (Fig. 7E, F). Spatial variations in soil respiration in different vegetation types have been ascribed to differences in soil C and N contents in a number of studies [8], [15], [16]. In contrast with other vegetation types, the *Phragmites australis* community likely possesses a higher organic C accumulation capability in Jiuduansha wetland [61]. The significant positive correlation between soil respiration rate and total N content can be explained by the direct dependence of plant growth and root activities and indirect dependence of aboveground net primary productivity (ANPP) on soil N availability [16], [19]. Luo et al. [21] also found that spatial variation in soil respiration was mainly caused by differences in total N and plant biomass among the six forest sites in different primary succession stages.

Although SOC is also expected to be a major carbon supply to the soil microbial respiration, soil respiration was not correlated with SOC in this study. Previous researchers also found that soil respiration rates were not significantly correlated to SOC in other ecosystems [6], [57]. It is probably noted that when the nutrient content is small, and microbial decay of soil organic matter is consequently small, so the relationships between soil respiration and organic carbon was not clear [57]. In addition, our results also showed higher total C, total N, and SOC in vegetated soil patches than bare soil patches, thereby, leading to much larger soil respiration from the soil beneath plant cover than from bare soil. Such a finding was mostly confirmed by many previous researches [7], [8]. Hence, the models of soil respiration should consider the patchy distribution of vegetation type, since it alters the characteristics of substrate quality and quantity, soil characteristics, and microbial activities, resulting in the spatial and temporal variations of soil respiration.

## Conclusions

On the diurnal timescale, daytime soil respiration varied correspondingly with net photosynthesis, and there were positive correlations between soil respiration and net photosynthesis at the *Phragmites australis* site. On the seasonal timescale, there were exponential correlations between soil respiration and soil temperature, and the fitted *Q*_10_ values varied among different vegetation types. Additional, there were significant linear relationships between soil respiration rates and shoot biomass and root biomass, respectively, during the early and peak growing season. At the field scale, the mean monthly soil respiration was positively correlated with shoot and root biomass, total C, and total N, while significant correlation was not found between soil respiration and litter biomass and SOC among the three vegetation patches. Our results suggest that vegetation patchiness at a field scale might have a large impact on ecosystem-scale soil respiration. Therefore, modeling soil respiration should not only take into account the simple soil temperature and moisture relationships, but also incorporate the patchy distribution of vegetation type as they affect the spatial and temporal variation of soil respiration.
